# Sub-25 nm Inorganic and Dielectric Nanopattern Arrays on Substrates: A Block Copolymer-Assisted Lithography

**DOI:** 10.1021/acsomega.1c05124

**Published:** 2021-12-16

**Authors:** Tandra Ghoshal, Nadezda Prochukhan, Michael A. Morris

**Affiliations:** School of Chemistry, AMBER and CRANN, Trinity College Dublin, Dublin D02 AK60, Ireland

## Abstract

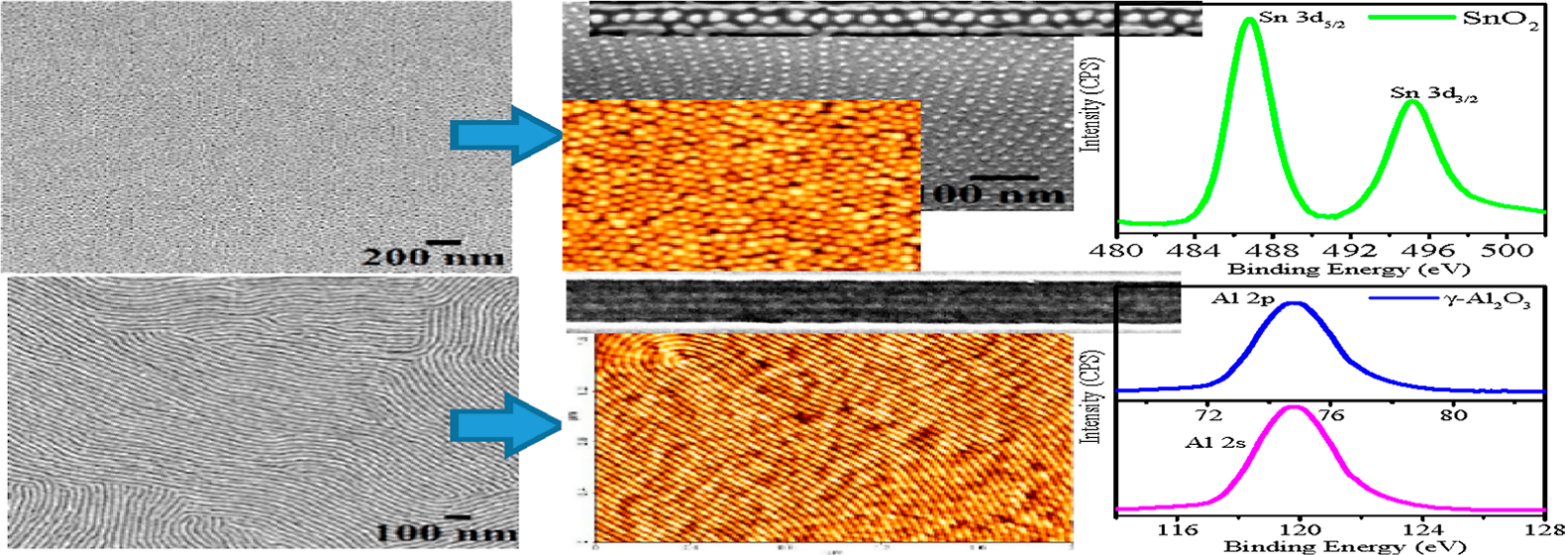

A range of well-ordered inorganic (antimony, tin, and tungsten oxide) and dielectric (silica, alumina, and hafnia) nanoparticles and nanowire array patterns are created on substrates by a low-cost block copolymer (BCP) approach. A cylindrical-phase PS-*b*-PEO BCP is used as a template with hexagonally ordered perpendicular or parallel orientation of PEO cylinders. The solvent annealing parameters such as solvents, temperature, time, and so forth are optimized to achieve the desired patterns. An established BCP in situ inclusion protocol is utilized to achieve the material nanopatterns by spin coating the respective precursor ethanolic solution on the template followed by UV/ozone treatment for oxide conversion and polymer removal. Furthermore, the precursor solution concentrations and stirring times are calibrated to achieve isolated, well-ordered, and uniform-diameter and -thickness nanoparticles and nanowires. All of the material nanopatterns are mimicking the parent BCP nanopatterns. The phases of all of the nanopatterns are determined by X-ray photoelectron spectroscopy. The inorganic and dielectric nanopattern arrays are patterned on a graphoepitaxial substrate for device application.

## Introduction

Large-scale arrays of nanostructures on substrates are used in many applications, including photonic,^1^ electronic,^2^ sensor,^3^ optoelectronic,^4^ microreactor,^5^ piezoelectric,^6^ porous filtration membrane,^7^ energy conversion,^8^ and energy storage devices.^9^ To achieve the regular nanostructure arrays, different fabrication techniques including lithography, nano-imprinting, and self-assembly have been developed. Block copolymer (BCP) self-assembly-based nanolithography^10,11^ can spontaneously generate periodic arrays of microdomains on substrates in the thin-film form with versatile morphology and nanoscale feature size in the range of 10–100 nm and has been widely recognized as a viable alternative or complementary approach to conventional photolithography.^12,13^ It can encounter the continuous demanding shrinkage of feature size for the electronic devices and the technologies in a cost-effective way. Thus, BCP-based nanolithography has been directed as one of the most important next-generation lithography techniques in the International Technology Roadmap for Semiconductors due to its magnificent benefits such as high throughput, low cost, and compatibility with current nanolithography streamline.^12^

The self-assembled di-BCP film can be used as an on-chip etch mask or a template, and material patterns can be produced by selective removal of one copolymer block and/or selective inclusion of chemical components to a chosen block with subsequent processing.^14,15^ Additionally, there is significant additional potential for the oxides as they covered almost all aspects of material science and physics in areas including properties like superconductivity, ferroelectricity, magnetism, and more. This is due to their two unique characteristics: variation in valence states and oxygen vacancies.^16^ We have reported the formation of inorganic oxide nanodots and nanowire patterns using selective inclusion of inorganics into the modified microphase-separated cylindrical phase PS-*b*-PEO thin films.^17,18^ We have previously focused on the formation of binary or ternary semiconductor nanopatterns by the in situ inclusion method.^19^ In this report, we have extended the idea of the formation of material nanopatterns for other inorganic oxide semiconductors and dielectrics. Dielectric materials have been extensively investigated as gate materials for microelectronic devices to reduce leakage currents in the miniaturization of modern devices.^20,21^ Also, they can be used in optical coatings such as interference filters and anti-reflection coatings.^22,23^

In this context, different semiconductors [antimony(III) oxide, tin(II) oxide, and tungsten(IV) oxide] and dielectrics (silica, alumina, and hafnia) nanostructure arrays were explored. Spun-cast followed by solvo-thermal treatment was applied to generate BCP patterns with vertically oriented cylindrical microdomains through the formation of solvent fronts and/or alteration of interfacial chemistry. Different techniques such as sequential infiltration synthesis utilizing the atomic layer deposition method allow the growth of materials from volatile precursors for selective binding of the precursor to one domain of the BCP system. However, the method, generally relatively complex, involves expensive fabrication equipment and is limited in the material sets that can be used.^24−26^ Herein, a simple, cost-effective method is utilized to create material patterns in terms of identical size/shape and regularity with each individuals of the same compositions. The modified BCP template is used as a host for inorganic salt inclusion, and subsequent UV/ozone treatment removes both the polymer and converts the salt to oxides. We have also generated the inorganic and dielectric nanopatterns on graphoepitaxial substrates, an essential measure for device applications. The BCP and other materials were reported to form within channels by different methods,^27−30^ but in this report, a similar methodology is followed to generate array patterns within different channel widths and lengths. Spectroscopic and microscopic techniques reveal the formation of uniform sized arrays of oxide semiconductors and dielectrics of nanoparticles and nanowires.

## Results and Discussion

**Figure 1:**
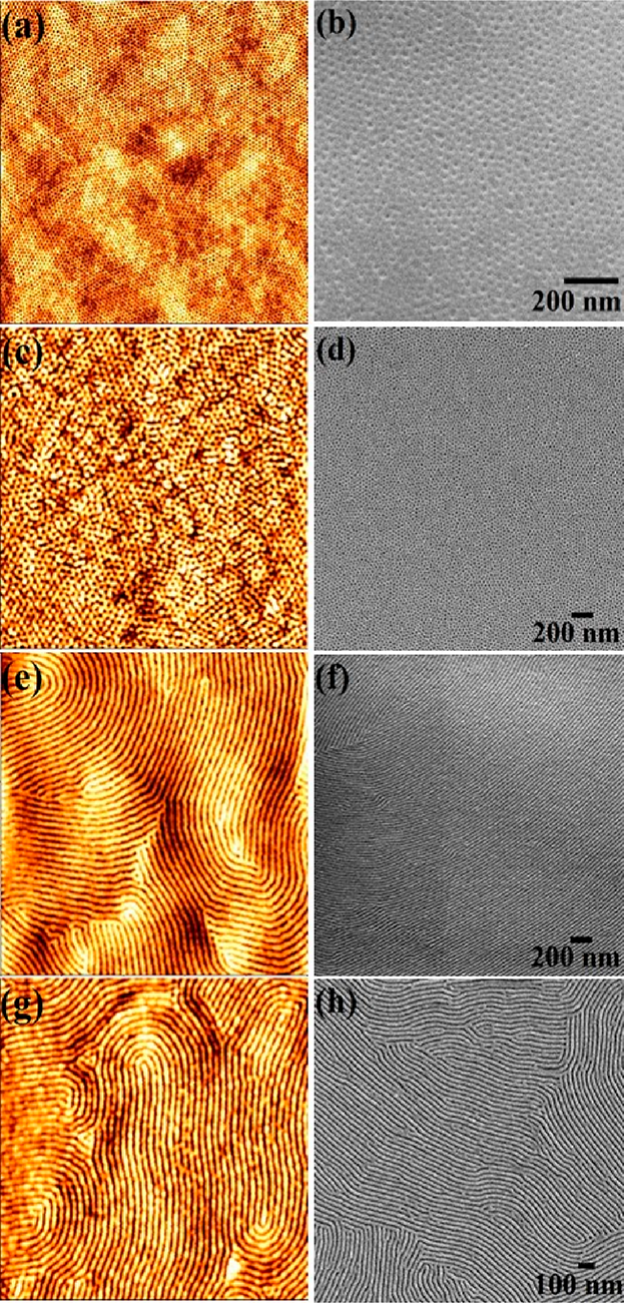
(a,e,b,f) Topographical AFM and SEM images of PS-*b*-PEO (42k–11.5k) after solvent annealing in toluene/water and toluene for 1 h at 50 and 60 °C, respectively. (c,g,d,h) AFM and SEM images of the respective BCP patterns after ethanol treatment at 40 °C for 15 and 17 h, respectively. AFM images (a,c,e,g) scale bar: 2 × 2 μm.

A cylindrical phase BCP, PS-*b*-PEO (42k–11.5k) with PEO as the minority cylinder forming microdomain block, was used. A solvent-mediated annealing was used to achieve the microphase-separated BCP nanopatterns forming PEO cylinders inside the PS matrix. The processing parameters such as annealing solvents, temperature, and time were carefully controlled to achieve the desired structural arrangement and orientation of the PEO cylinders. To achieve the perpendicular orientation of the PEO cylinders, the spun-cast film was exposed to mixed toluene/water solvents (in separate reservoirs) at a temperature of 50 °C for 1 h under vacuum. Note that the vacuum is necessary during the entire annealing process to avoid moisture on the film surface causing dewetting. The morphological evolution with different experimental parameters was described previously.^17−19,31,32^Figure 1a,b shows the representative non-contact mode topographical atomic force microscopy (AFM) and scanning electron microscopy (SEM) images of the BCP after the solvent exposure, respectively. The large-area AFM image indicates ordered arrangements without dewetting. The SEM (Figure 1b) image also indicates similar structural arrangements of PS and PEO microdomains despite their similar densities and average atomic number. The film surface is with an uniform level with a thickness of around 40 nm, as measured using an ellipsometer. The average measured center-to-center distance between adjacent PEO cylinders is 42 nm with a diameter of 19 nm. The orientation of the PEO cylinders being parallel to the substrate surface can be altered by switching the annealing solvent to toluene with an operating temperature of 60 °C for 1 h (Figure 1e,f). AFM and SEM images confirm the parallel orientation of PEO cylinders to the substrate surface with similar center-to-center spacing and PEO cylinder diameter. The fingerprint patterns are stretched with a persistent length up to 2 μm. The SEM image confirms long-range ordered line/space patterns.

As described in our previous reports that etch and/or modification of cylindrical PEO microdomains is an essential prerequisite to incorporate metal ions into one of the BCP blocks in order to fabricate inorganic nanopatterns referred as an “activation step.” This is realized by dipping the patterned substrate in anhydrous ethanol at 40 °C for different time periods for dots and lines/space patterns. The immersion time is increased from 15 h for holes to 17 h for line patterns, probably due to more exposed PEO surface areas. The structural periodicity and dimensions remained the same after ethanol treatment, as revealed by AFM and SEM images in Figure 1c,d,g,h, respectively. The ethanol exposure discloses an increment in the phase contrast without affecting the long-range order, as seen from all images.

The cylinder-to-cylinder spacings and the PEO cylinder diameters remained unaltered. No measurable change in film thickness was observed. The film is strongly adhered to the substrate surface as no slitting or defects is noticed on the interface after the ethanol treatment. This implied the applicability of the modification process for both hole and line/space patterns.

The challenges and limitations of the etch protocol for pattern transfer onto the substrates by conventional BCP lithography restrict their size and quality factors because of its use as a soft mask generally achieved by selective removal of one block and subsequent use of the other. Alternatively, diblock copolymer (DBCP) nanopatterns can be used to create a hard mask (i.e., a material with very high etch resistance compared to the substrate). Previous studies suggest that dielectrics (SiO_2_, Al_2_O_3_, and Si_3_N_4_), various metal oxides (Fe_2_O_3_ and NiO), and metals (Cr and Ni) are generally preferred to be used as a hard mask for high-aspect-ratio silicon substrate patterning. Our idea here is that to fabricate on-chip various metal oxides and dielectric hard mask nanoparticles/line patterns for their use to create semiconductor nanopatterns of interest. In this regard, our established BCP in situ inclusion protocol is explored further, and the strategy is modified for several materials and patterns on flat and graphoepitaxial substrates.

**Figure 2:**
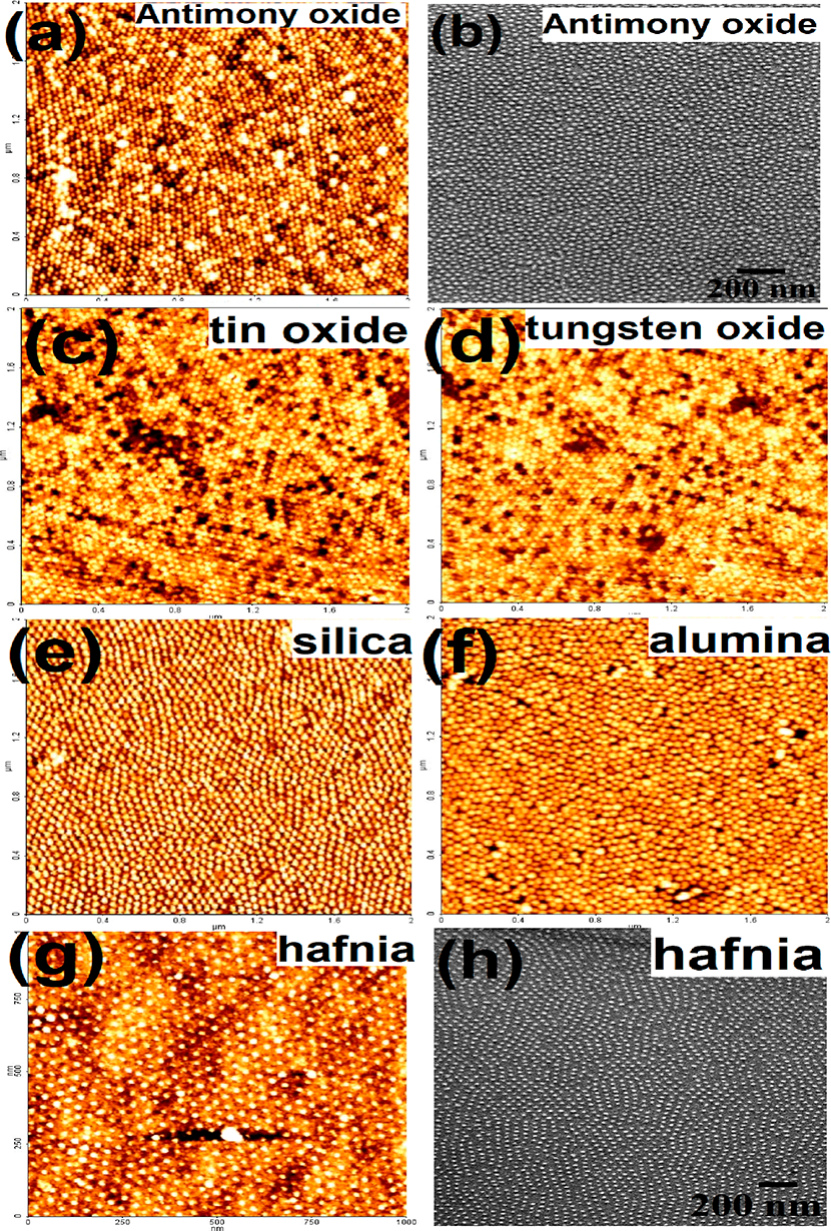
(a,c,d,e,f,g) AFM images of antimony, tin, tungsten oxides, silica, alumina, and hafnia nanoparticle arrays after spin coating different concentrations of respective precursor ethanolic solution followed by UV/ozone treatment. (b,h) SEM images of antimony oxides and hafnia nanoparticles array. AFM images (a,c,d,e,f) scale bar: 2 × 2 μm. (g) 1 × 1 μm.

The nanohole polymer templates are utilized to form ordered nanoparticle arrays. Figure 2 shows the AFM and SEM images of different inorganic oxides and dielectric nanoparticle arrays prepared using different precursors with varying solution (in anhydrous ethanol) concentrations. The precursor ethanol concentrations varied to avoid any overfilling or missing patterns. For different precursors used, the precursor solution concentrations and stirring times are different depending on the rate of hydrolization and their ability to dissolve in ethanol at room temperature. Figure 2a,b shows ordered antimony oxide nanoparticle array using 0.05 wt % of SbCl_3_-ethanolic solution spin-coated onto the polymer template followed by UV/ozone treatment. The disparity of diameter (22 ± 3 nm) and height (6 ± 2 nm) of the nanoparticles throughout the wafer is evident. Similarly, the AFM images in Figure 2,d depict better uniformity in their diameter and heights for tin oxides and tungsten oxide nanoparticle arrays using 0.08 and 0.05 wt % of SnCl_4_-ethanolic and WCl_4_-ethanolic solution, respectively. The measured mean diameters are 24 and 22 nm, and the measured height is 6 nm for tin and tungsten oxides, respectively. All of the inorganic semiconductor oxides examined maintain the original center-to-center nanoparticle spacing (42 nm). The higher precursor concentration is required for tin oxide due to the water content in the precursor. Similarly, less solution stirring time (15 min) is sufficient for the formation of tin oxides before spin coating in comparison to other oxides (20 min) due to their hygroscopic nature.

Similar processing is followed to generate dielectric nanoparticle array. The AFM images in Figure 2e–g display ordered arrays of silica, alumina, and hafnia using 0.05, 0.08, and 0.05 wt % of respective-ethanolic solution spin-coated onto the polymer template followed by UV/ozone treatment. Compared to other oxides (20 min), less stirring time (10 min) is required for the formation of silica nanoparticles because the precursor reacts easily with atmospheric moisture, resulting in silica clusters on the film surface. The diameter of the nanoparticles is 22, 24, and 20 nm for silica, alumina, and hafnia, respectively. The nanoparticles are of uniform diameter for silica and alumina, but a broad diameter distribution (±3 nm) and missing patterns are noticed for hafnia, as described in AFM and SEM images (Figure 2g,h). The heights of the nanoparticles are in the range of 4–6 nm. It is noticed that a slight variation in the precursor concentrations and stirring time greatly influences the quality and ordering of the nanoparticle formation. However, the precursor concentrations and stirring times are carefully optimized but need to be controlled more minutely. As the experiments are performed in ambient air atmosphere, the precursor solution stirring time and the rate of oxide formation should vary with temperature, humidity, and so forth. This is the reason why a broad diameter distribution ∼3 nm or 14% is noticed for few of the materials. The process of metal ion inclusion into the porous template is rapid, achieved during spin coating predominantly because of the selective affinity of PEO with the ionic solution and hindrance of any ion into the hydrophobic PS. Thus, it can be concluded that the same strategy is applicable for generating a range of inorganic semiconductor oxides and dielectrics nanoparticle arrays. Essentially, the center-to-center nanoparticle distance remained the same as the parent BCP nanopatterns.

**Figure 3:**
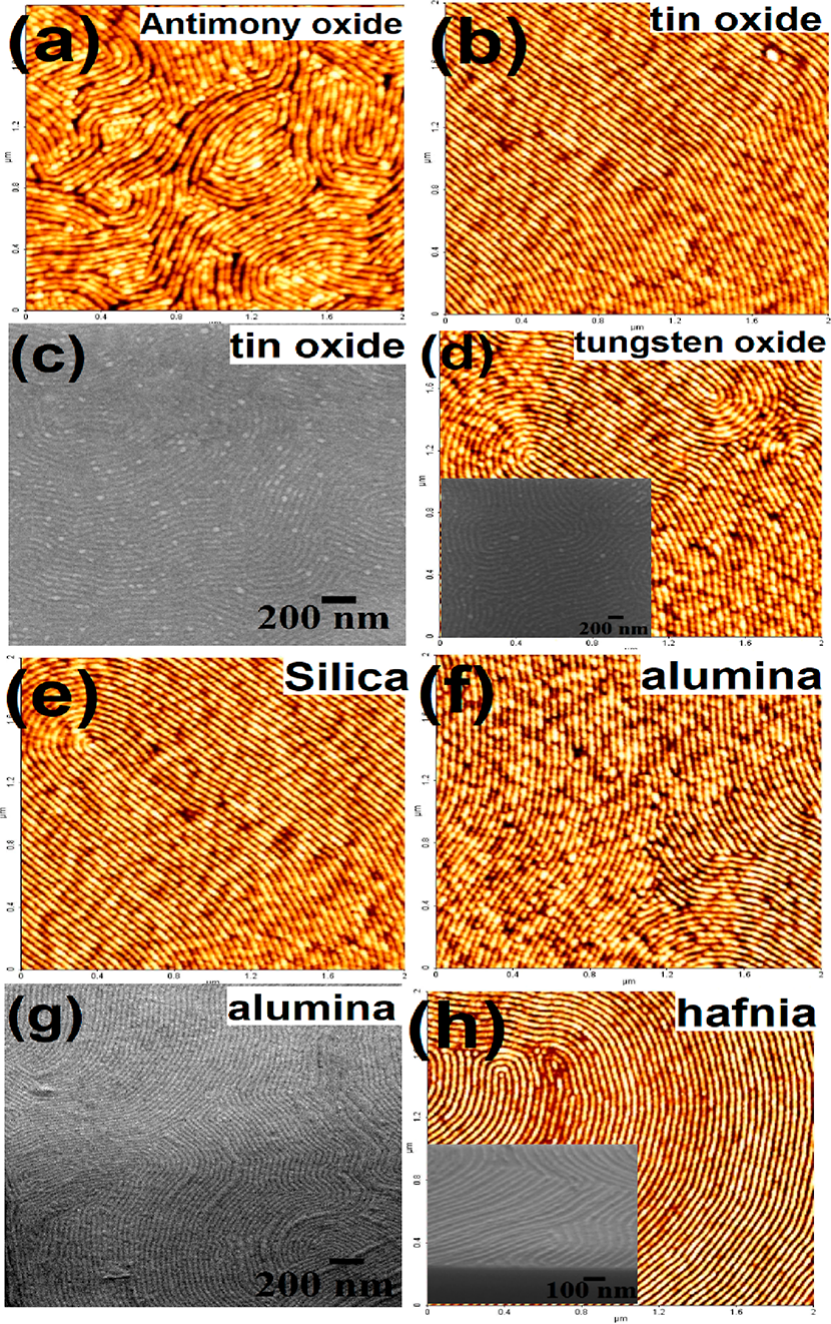
(a,b,d,e,f,h) AFM images of antimony, tin, tungsten oxides, silica, alumina, and hafnia nanowire arrays after spin coating different concentrations of respective precursor ethanolic solution followed by UV/ozone treatment. (c), (d, inset), (g), (h, inset) SEM images of tin oxide, tungsten oxide, alumina, and hafnia nanowire arrays. AFM images (a,b,d,e,f,h) scale bar: 2 × 2 μm.

Similarly, a range of inorganics and dielectrics are explored to generate horizontal well-ordered nanowire array patterns on the substrate. Figure 3a shows ordered antimony oxide nanowire array using 0.2 wt % of SbCl_3_-ethanolic solution spin-coated onto the polymeric nanoporous wire template followed by UV/ozone treatment. The diameter and height of the nanowires are 22 and 4 nm, respectively. Compared to nanoparticle arrays, nanowires are well ordered and are with uniform diameter. Few of the scattered nanoparticles is noticed on top of the nanowires probably because of residual precursor solution during spin coating and further oxidation. A similar kind of particle formation on top of the nanowires is realized for tin oxide and tungsten oxide nanowire arrays formed by using 0.5 and 0.2 wt % precursor solution, as shown in Figure 3b–d, respectively. All of the nanowires are isolated, continuous, well-ordered, and with uniform diameter and thickness. The diameter and height of the nanowires are 20 and 4 nm for both of the oxides.

Similar processing is followed to generate dielectric nanowire arrays. The AFM images in Figure 3e,f,h display ordered arrays of silica, alumina, and hafnia using 0.05, 0.08, and 0.05 wt % of respective precursor ethanolic solution spin-coated onto the polymer template followed by UV/ozone treatment. Compared to hafnia nanowires, thicker diameter silica and alumina nanowires were realized. The SEM image, as shown in Figure 3g, also depicts thicker diameter alumina nanowire in few of the places. The alumina and silica precursors were oxidized more easily than hafnia by absorbing atmospheric moisture during stirring. Frequent nanoparticle formation (∼22 nm diameter) was noticed on top of the alumina nanowires because of deposition and further oxidation of the particulates formed within the precursor solution. The diameter of the nanowires is 20, 22, and 18 nm for silica, alumina, and hafnia, respectively. The height measured is in the range of 3–4 nm for all of the oxides. Cross-sectional SEM image in the inset of Figure 3h also reflects thin hafnia nanowires. Most of the nanowires are isolated, continuous, well-ordered, and with uniform diameter and thickness. Few broken nanowires were realized for hafnia. A broad diameter distribution (±3 nm) and infused nanowires were noticed for alumina.

The mechanism for the “activation step” by ethanol treatment and the metal ion inclusion process were described in detail in previous reports.^17−19,31^ After solvent annealing, the film surface is PS rich, and a wetting layer exists. A slow structural change occurs after ethanol exposure that removes this thin wetting layer. The similarity of the chemical structures of PEO monomers [(CH_2_CH_2_O)−] and ethanol molecules (H–CH_2_CH_2_O–H) is an important criterion to dissolve PEO fragments, allowing crystallization of the PEO. When the film was taken out from ethanol, the PEO molecules were trying to separate from the solution but cannot because of this similarity; as a result, the PEO chains are frustrated and have no choice to form a thin crystalline layer. The PEO-activated sites were considered as sorbitive cylinders because hydrophobic nature of PS excludes any probability of the metal ion inclusion into the PS matrix. Affinity of PEO with cations allows rapid absorption of the metal ions through either intra- or intermolecular coordination via electron donation from the PEO block oxygen atoms. The tendency toward multiple bindings would be favored by densely packed crystalline PEO chains necessary for effective inclusion of the metal species. If PEO cylinders were completely removed, it would be highly unlikely that significant metal uptake would occur because the PS matrix would be hydrophobic, and the concentration of metal is rather low.

The effects of precursor solution concentrations on the formation of alumina nanowire arrays were examined (see the Supporting Information). Figure S1a,b shows the SEM images of alumina nanowires using 0.1 and 0.06 wt % of concentrated precursor ethanol solution spin-coated onto the polymer template followed by UV/ozone treatment. A large-area view shows nanowires with bigger diameter, but frequent particulate deposition was noticed on top of the nanowires (Figure S1a). With less concentrations used, non-uniform diameter-broken nanowires were realized (Figure S1b).

**Figure 4:**
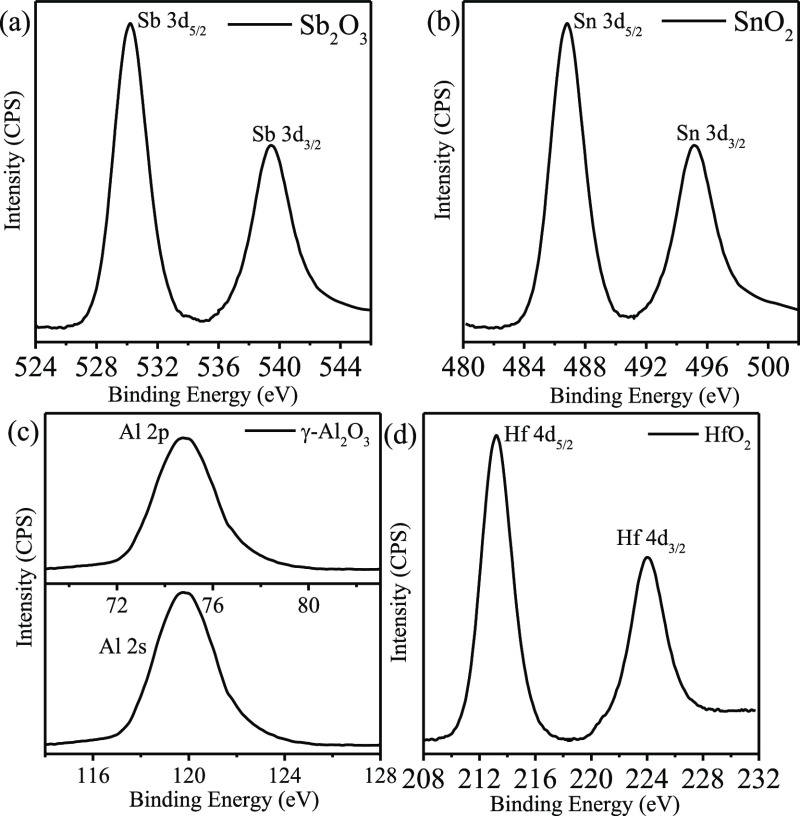
High-resolution (a) Sb 3d, (b) Sn 3d, (c) Al 2p and 2s, and (d) Hf 4d XPS spectrum for the as-prepared inorganic oxide, dielectric nanoparticle, and nanowire array.

It is necessary to identify the chemical compositions and phases of the as-fabricated inorganic and dielectric oxide nanoparticles and nanowire arrays after UV/Ozone treatment for their use in different potential applications confirmed by X-ray photoelectron spectroscopy (XPS) analysis. Figure 4a shows the high-resolution Sb 3d spectrum, reflecting the oxidation state of the as-prepared antimony oxide nanoparticles and nanowires. As the binding energy values of Sb 3d_5/2_ and O 1s both are in the 530 eV regime, it is very difficult to interpret their contributions to the total peak area values. This was carefully optimized from the intervention of software and high-resolution Sb 3d spectrum. The two peaks from atomic orbits of 3d_3/2_ and 3d_5/2_ have a constant distance of 9.3 eV, and the ratio of these two peak areas is 2:3, reflecting the Sb_2_O_3_ phase of antimony oxide consistent with the reported values.^33,34^ The phase of the as-prepared tin oxide nanoparticles and nanowire arrays is determined from the Sn 3d XPS spectrum (Figure 4b) showing the binding energy peaks of Sn 3d_5/2_ and Sn3d_3/2_ at 486.7 and 495.1 eV, respectively, which correspond to the typical oxidized state of Sn^4+^ in the SnO_2_ phase.^35,36^ The oxidation state for tungsten oxide is determined was WO_3_ from W 4f spectrum (not shown). The W 4f_7/2_ and W 4f_5/2_ doublet present at 35.6 and 37.7 eV (with a peak separation of 2.1 eV and the intensity ratio of 3:4) corresponds to W^6+^ ions.^37,38^ Survey spectra for all of the semiconductors reveal pure phase of oxides without any residual polymer.

The phases of the dielectrics are also revealed by the corresponding high-resolution XPS spectra. The as-prepared phase of silicon oxide nanoparticles and nanowire arrays corresponds to silicon dioxide, as revealed by the Si 2p peaks at 100.5 and 102.3 eV (not shown).^39,40^ The phase of aluminum oxide is revealed by Al 2p and Al 2s spectra, as shown in Figure 4c. The peaks positioned at 74.8 and 119.7 eV for 2p and 2s, respectively, correspond to the γ-Al_2_O_3_ phase.^41^ The stability of the silica nanoparticles and alumina nanowire patterns is checked by annealing them at 1000 °C for 1 h. The patterns remain unaltered by annealing, but the diameter of the nanofeatures reduced by ∼2 nm due to high-temperature densification (see the Supporting Information). Similarly, the as-prepared hafnium oxide nanofeatures show that Hf 4d_5/2_ and Hf 4d_3/2_ peaks at 213.1 and 223.8 eV correspond to Hf(IV) in HfO_2_.^42^

**Figure 5:**
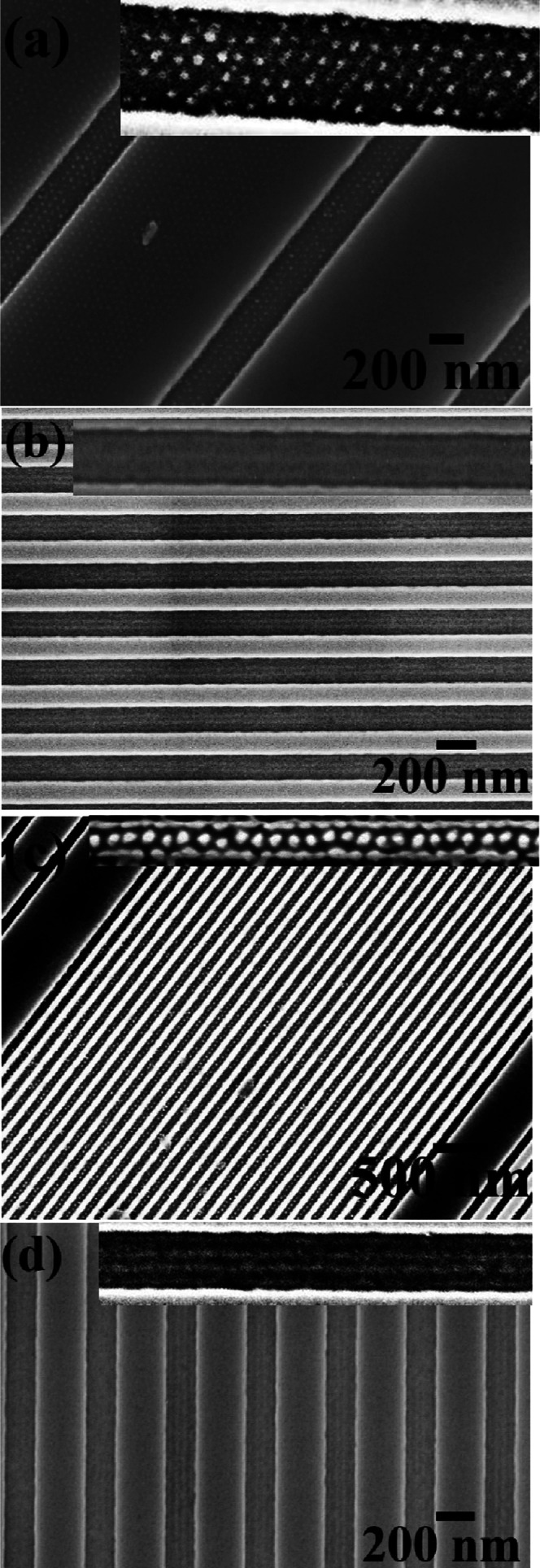
(a–d) Ordered arrays of tin oxide and alumina nanoparticles and line patterns within channel widths of 240, 160, 90, and 240 nm, respectively.

In this study, we also examine whether the inorganic and dielectric nanopatterns could be created on graphoepitaxial substrates, an essential measures for device applications. In this regard, a graphoepitaxial substrate of different channel widths was used to generate ordered microphase-separated PS-*b*-PEO BCP nanopatterns. A 7 nm thick silica layer-coated Si substrate with 50 nm deep topographically defined patterns of SiN sidewall was used as a substrate. The concentrations of the BCP-toluene solution for spin coating was calibrated to 0.5 wt % to avoid any overfilling within the channels. Similar process steps for generating materials on a flat substrate were followed to achieve the ordered material nanopatterns within trench. Figure 5a,b shows ordered arrays of tin oxide nanoparticles and line patterns within channel widths of 240 and 160 nm, respectively. Six arrays of particles were realized, whereas two arrays of dots attached to the sidewalls of the trench reveal that this specific trench width is minimal to achieve six arrays. Well-ordered three arrays of line patterns were realized along the entire channel length. Compared to dots, lines were formed along the sidewalls and inside the trench. Note that similar precursor solution concentrations were used to achieve tin oxide particles and lines of diameter of 22 and 18 nm, respectively. Aluminium oxide particles and line pattern arrays were also formed after spin-coating 0.08 wt % of precursor ethanolic solution followed by UV/ozone treatment within channel widths of 90 and 240 nm, respectively (Figure 5c,d). An array of nanoparticles at the center of the trench is observed, whereas three arrays of nanowire arrays were formed with diameters of 20 and 18 nm, respectively. All of the nanowires formed were continuous and of regular diameter along the trench. Essentially for all of the nanoparticles and nanowires, the center–center spacing remained the same as 42 nm.

## Conclusions

A cylindrical phase PS-*b*-PEO BCP is explored to achieve the microphase-separated hexagonally ordered perpendicular or parallel orientation of PEO cylinders inside the PS matrix. A toluene/water mixed solvent is optimized to achieve the hole patterns, whereas toluene is sufficient to achieve the line patterns though slightly higher temperature is required. A range of well-ordered nanoparticles and nanowire array patterns of inorganics (antimony, tin, and tungsten oxide) and dielectrics (silica, alumina, and hafnia) were generated on the substrate. An established BCP in situ inclusion protocol was utilized to achieve the material nanopatterns where respective precursor ethanolic solution was spin-coated onto an etched/modified BCP template followed by UV/ozone treatment. For different precursors used, the precursor solution concentrations and stirring times are calibrated depending on the rate of hydrolization and their ability to dissolve in ethanol at room temperature. All of the nanoparticles and nanowires are isolated, well-ordered, and with uniform diameter and thickness with same center–center spacing as parent BCP nanopatterns. The phases of all of the nanopatterns were determined by XPS as Sb_2_O_3_, SnO_2_, WO_3_, SiO_2_, γ-Al_2_O_3_, and HfO_2_. The inorganic and dielectric nanopattern arrays can be created on a graphoepitaxial substrate showing their applicability for devices. This is an alternative approach, which avoids complicated lithographic steps at a lower cost.

## Experimental Section

### Preparation of Oxide Nanoparticles and Nanowire Arrays by BCPs

PS-*b*-PEO was purchased from Polymer Source and used without further purification (number-average molecular weight, *M*_n_, PS = 42 kg mol^–1^, *M*_n_, PEO = 11.5 kg mol^–1^, and *M*_w_/*M*_n_ = 1.07, where *M*_w_ is weight-average molecular weight). All of the precursors were purchased from Merck and used without further purification. Highly polished single-crystal silicon ⟨100⟩ wafers (p-type) with a native oxide layer were used as a substrate without any attempt to remove the native oxide layer. Ultrasonication of the substrates in acetone and toluene separately for 30 min removes dirt and grease and so forth and dried immediately by nitrogen stream. The DBCP was dissolved in toluene by stirring at room temperature to yield a 1 wt % solution for at least 12 h prior to use. PS-*b*-PEO thin films were spin-coated onto silicon substrates at 3000 rpm for 30 s using a SCS G3P-8 spin coater. The films were exposed to toluene or toluene/water mixed vapor placed at the bottom of a closed vessel kept at a temperature of 50 and 60 °C for 1 h to induce microphase separation through the required chain mobility. Both horizontally and vertically aligned PEO cylinders were realized at different experimental conditions. PEO microdomains are partially etched and/or modified by dipping the film in anhydrous alcohol at 40 °C for 15–18 h. The films were taken out from alcohol and dried immediately after the desired time. For the fabrication of oxide nanoparticles and nanowires, different concentrations of salts were dissolved in anhydrous alcohol, stirred for different times, and spun-cast onto the modified films. The salts used for the oxides were antimony(III) chloride (SbCl_3_), tin chloride pentahydrate (SnCl_4_, 5H_2_O), tungsten(IV) chloride (WCl_4_), tetraethyl orthosilicate, aluminium nitrate nonahydrate [Al(NO_3_)_3_, 9H_2_O], and hafnium(IV) chloride (HfCl_4_). UV/ozone processing oxidizes the precursor and removes the polymer. The concentrations of the precursor solutions varied to achieve the optimum uniform monodispersed nanoparticles and continuous horizontal nanowire arrays, and the subsequent effects were examined. The attachment of the nanoparticle arrays with the substrate was verified by thermal treatment at 1000 °C for 1 h.

### Characterizations

Surface morphologies of the nanostructured thin films were analyzed with scanning probe microscopy (Park systems, XE-100) in the tapping mode and SEM (FEG Quanta 6700 and Zeiss Ultra Plus). The film thicknesses were measured using an optical ellipsometer (Woolam M2000) at a minimum of five different locations on the sample. A two-layer β-spline model (SiO_2_ + BCP) was used to simulate the experimental data. XPS experiments were performed with an Al Kα X-ray source operating at 72 W.

## Supporting Information Available

SEM images of alumina nanowires using different concentrations of the precursor solution and SEM images of silica and alumina nanowires after annealing (PDF)
