# Supplementary material for: Sub-25 nm Inorganic and Dielectric Nanopattern Arrays on Substrates: A Block Copolymer-Assisted Lithography
Source: ACS Omega. 2021 Dec 16;6(51):35738–44. doi: 10.1021/acsomega.1c05124 (PMC8717533; doi:10.1021/acsomega.1c05124)
Supplement: Supplementary file 1 — ao1c05124_si_001.pdf [file ao1c05124_si_001.pdf]

Sub-25 nm inorganic and dielectric nanopattern arrays on substrates: A block copolymer assisted lithography

Tandra Ghoshal,* Nadezda Prochukhan, Michael A. Morris*

School of Chemistry, AMBER and CRANN, Trinity College Dublin, Dublin, Ireland, D02 AK60

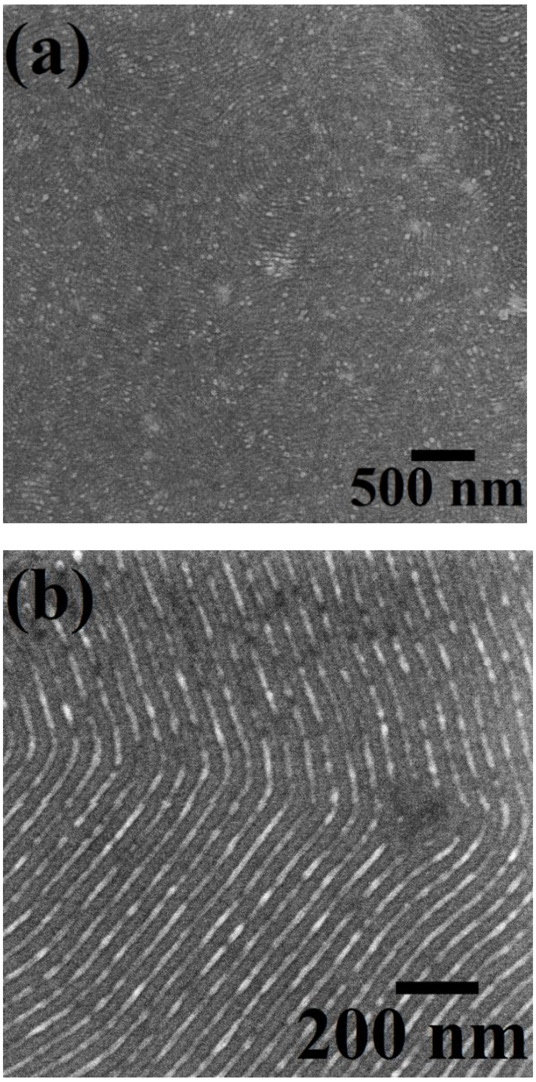

Figure S1 SEM images of alumina nanowires using (a) 0.1 and (b) 0.06 wt% of concentrated precursor-ethanol solution spin coated onto the polymer template followed by UV/ozone treatment.

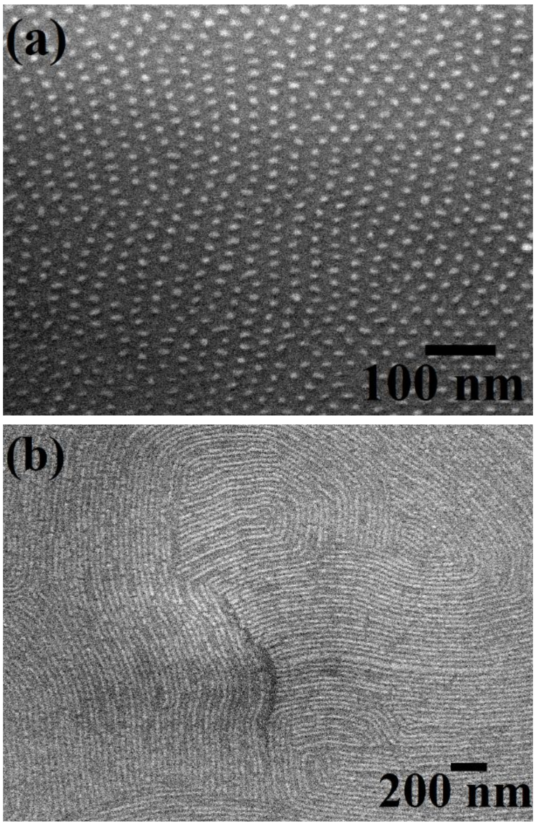

Figure S2 (a) Silica nanoparticles and (b) alumina nanowires patterns after annealing at 1000 ℃ for 1 h.
